# Dissecting the genetic basis of focal cortical dysplasia: a large cohort study

**DOI:** 10.1007/s00401-019-02061-5

**Published:** 2019-08-23

**Authors:** Sara Baldassari, Théo Ribierre, Elise Marsan, Homa Adle-Biassette, Sarah Ferrand-Sorbets, Christine Bulteau, Nathalie Dorison, Martine Fohlen, Marc Polivka, Sarah Weckhuysen, Georg Dorfmüller, Mathilde Chipaux, Stéphanie Baulac

**Affiliations:** 1 Sorbonne Université, UPMC Univ Paris 06, UMR S 1127, Paris, France; 2 INSERM, U1127, Paris, France; 3 CNRS, UMR 7225, Paris, France; 4 Institut du Cerveau et de la Moelle épinière (ICM), Hôpital Pitié-Salpêtrière-47, bd de l’hôpital, 75013 Paris, France; 5 INSERM UMR 1141, Hôpital Robert-Debré, 75019 Paris, France; 6 Faculté de Médecine Denis Diderot, Université Paris 7, Paris, France; 7 Service d’Anatomie et de Cytologie Pathologiques, Hôpital Lariboisière, APHP, 75010 Paris, France; 8 Department of Pediatric Neurosurgery, Rothschild Foundation Hospital, 75019 Paris, France; 9 Department of Neurology, University Hospital Antwerp, Antwerp, Belgium

**Keywords:** Neurogenetics, Epilepsy-associated focal cortical dysplasia, mTOR pathway, Somatic variant, Brain mosaicism

## Abstract

Genetic malformations of cortical development (MCDs), such as mild MCDs (mMCD), focal cortical dysplasia (FCD), and hemimegalencephaly (HME), are major causes of severe pediatric refractory epilepsies subjected to neurosurgery. FCD2 are characterized by neuropathological hallmarks that include enlarged dysmorphic neurons (DNs) and balloon cells (BCs). Here, we provide a comprehensive assessment of the contribution of germline and somatic variants in a large cohort of surgical MCD cases. We enrolled in a monocentric study 80 children with drug-resistant epilepsy and a postsurgical neuropathological diagnosis of mMCD, FCD1, FCD2, or HME. We performed targeted gene sequencing ( ≥ 2000X read depth) on matched blood–brain samples to search for low-allele frequency variants in mTOR pathway and FCD genes. We were able to elucidate 29% of mMCD/FCD1 patients and 63% of FCD2/HME patients. Somatic loss-of-function variants in the N-glycosylation pathway-associated *SLC35A2* gene were found in mMCD/FCD1 cases. Somatic gain-of-function variants in *MTOR* and its activators (*AKT3*, *PIK3CA*, *RHEB*), as well as germline, somatic and two-hit loss-of-function variants in its repressors (*DEPDC5, TSC1*, *TSC2*) were found exclusively in FCD2/HME cases. We show that panel-negative FCD2 cases display strong pS6-immunostaining, stressing that all FCD2 are mTORopathies. Analysis of microdissected cells demonstrated that DNs and BCs carry the pathogenic variants. We further observed a correlation between the density of pathological cells and the variant-detection likelihood. Single-cell microdissection followed by sequencing of enriched pools of DNs unveiled a somatic second-hit loss-of-heterozygosity in a *DEPDC5* germline case. In conclusion, this study indicates that mMCD/FCD1 and FCD2/HME are two distinct genetic entities: while all FCD2/HME are mosaic mTORopathies, mMCD/FCD1 are not caused by mTOR-pathway-hyperactivating variants, and ~ 30% of the cases are related to glycosylation defects. We provide a framework for efficient genetic testing in FCD/HME, linking neuropathology to genetic findings and emphasizing the usefulness of molecular evaluation in the pediatric epileptic neurosurgical population.

**Electronic supplementary material:**

The online version of this article (10.1007/s00401-019-02061-5) contains supplementary material, which is available to authorized users.

## Introduction

Focal cortical dysplasia (FCD) and hemimegalencephaly (HME) are malformations of cortical development (MCDs) representing the most common cause of neocortical childhood-onset seizures [4, 14]. Seizures associated with FCD/HME are often refractory to antiepileptic drugs, and require surgical resection of the epileptogenic zone, allowing direct access to the dysplastic brain tissue for research purposes [8]. FCDs account for 17% of the children epilepsy surgery population, representing a major medical burden [6]. The international league against epilepsy (ILAE) has proposed a consensus for classification of FCDs based on neuropathological findings [7, 33]. FCD1 refers to isolated lesions with dyslamination of the neocortex. FCD2 refers to isolated lesions characterized by cortical dyslamination and dysmorphic neurons (DNs) in type 2a or both DNs and balloon cells (BCs) in type 2b. The most severe end of the spectrum is HME characterized by the enlargement of all or part of one cerebral hemisphere, often exhibiting FCD2a/2b histological features. In addition, mild forms of MCD (mMCDs) are characterized by an excess of heterotopic neurons in cortical layer 1 (mMCD type 1) or by microscopic neuronal clusters or excess of heterotopic single neurons of normal morphology in deep white matter (mMCD type 2) [7, 38].

Because of the focal nature of FCD, a role for somatic mosaic variants has been proposed [40]. Indeed, brain somatic variants in genes of the mTOR (mechanistic target of rapamycin) pathway have been reported in patients with various MCDs, from small FCD to larger HME [9, 10, 19, 25–27, 31, 32, 34, 39]. Hyperactivation of the mTOR pathway has been shown in FCD2/HME brain specimens (see reviews [18, 24, 29]), as well as in cellular and animal models of focal cortical expression of mutant *MTOR* or focal deletion of *DEPDC5* [2, 16, 17, 27, 28, 34, 41]. The mTOR-signaling cascade plays a key role in the integration of various environmental signals to regulate cell growth, proliferation, and metabolism [23]. mTOR hyperactivation during neurodevelopment is assumed to cause the cellular hypertrophy observed in DNs and BCs. BCs are heterogeneous cell types and have been reported to express both neuronal and glial markers, while DNs express mostly Smi-32/Smi-311 neurofilament proteins [37, 43, 46]. However, it has never been proven that these pathological cells, underlying epileptogenesis, carry the disease-causing variants.

Here, we report clinical, neuropathological, and molecular findings in a large monocentric cohort of 80 epileptic children subjected to neurosurgery and diagnosed with a cortical dysplasia (FCD or HME) or a mMCD. We designed a pipeline to efficiently detect low-allele frequency brain variants by capture-based gene panel sequencing, and accurately assess the prevalence of brain somatic and germline variants. Single-cell microdissection was performed to determine the cell type of origin of the variants. Finally, we performed a correlation analysis between clinicopathological features and genetic findings.

## Methods

### Cohort recruitment and samples collection

We enrolled 80 children subjected to surgery for the treatment of drug-resistant epilepsy at the Rothschild Foundation Hospital (Paris, France) between 2015 and 2018. The inclusion criterion was a post-operative diagnosis of mMCD, FCD or HME assessed by two experienced neuropathologists (H.A.B., M.P.). We collected clinical information before and after surgery for each patient. Presurgical MRI were systematically reviewed by a specialized neuroradiologist and by a pediatric epileptologist neurosurgeon (G.D. or S.F.S.). Subjects without precisely delimited MRI-lesions (58/80) underwent SEEG and/or 18-fluorodeoxyglucose positron emission tomography scan (18FDG-PET) investigation co-registered with MRI to further delineate the seizures onset zone (SOZ). A cognitive evaluation was conducted by the neuropediatricians (C.B., N.D., M.F., M.C.). Autistic features were defined by impairments in social interaction and communication, restricted interests and repetitive behaviors. Presurgical evaluation and surgical procedures were performed as previously reported [12]. After surgery, brain specimens were formalin-fixed and paraffin-embedded (FFPE) by the neuropathology department, and an adjacent block was immediately frozen in liquid nitrogen for research purposes.

Neuropathological diagnoses were performed according to the ILAE guidelines [7]. FCD1 referred to isolated lesions with cortical dyslamination, and were classified as FCD1a when an excess of microcolumns was observed; FCD2a referred to cortical dyslamination and presence of DNs, and FCD2b referred to cortical dyslamination and presence of DNs and BCs. mMCD referred to surgical cases with blurred grey–white matter boundaries and excess of heterotopic neurons in cortical layer 1 (mMCD1) or in the deep white matter (mMCD2). Blood samples from patients and their parents (when available) were collected. Genomic DNA was extracted according to the standard procedures from blood samples and frozen brain sections or bulk tissue.

This study was approved by the ethical committee CPP Ile de France II (N° ID-RCB/EUDRACT-2015-A00671-48) and is registered on ClinicalTrials.gov (N° NCT02890641). Written informed consent was obtained from all participants (or their parents on behalf).

### Detection and validation of somatic variants

Hybrid capture sequencing targeting coding exons and exon-flanking junctions (10 bp) were performed (Roche NimbleGen SeqCapEZ Human custom panel). We used three updated versions of a panel comprising mTOR pathway and FCD candidate genes, including *SLC35A2*. Genes common to all versions are: *AKT3* (NM_005465), *DEPDC5* (NM_001242896), *MIOS* (NM_019005), *MTOR* (NM_004958), *NPRL2* (NM_006545), *NPRL3* (NM_001077350), *PIK3CA* (NM_006218), *PTEN* (NM_000314), *SEC13* (NM_183352), *SEH1L* (NM_001013437), *TSC1* (NM_000368), *TSC2* (NM_000548), *WDR24* (NM_032259), and *WDR59* (NM_030581). *BRAF* (NM_004333) was included in the two last versions of the panel, and *SLC35A2* (NM_005660) and *RHEB* (NM_005614) were included in the last panel only. A full list of the genes included in each version of the panel is available in Supplemental Data (Online Resource 1). Libraries were prepared according to standard protocols and sequenced on an Illumina NextSeq 500 sequencer (2 × 75 bp) at the iGenSeq sequencing facility of ICM. Bioinformatic analysis was conducted by Genosplice (www.genosplice.com) as previously described [32]. Germline variants were called according to GATK’s best practices using HaplotypeCaller. Somatic single nucleotide variants (SNVs) or small insertions/deletions (indels) were called with SAMtools-1–1 (mpileup) and an in-house developed bioinformatic pipeline [32]. Somatic variants were filtered for variant allele frequencies (VAFs) ≥ 1%, alternate coverage ≥ 10 reads, base quality score ≥ 25, and mapping quality score ≥ 50. Recurrent pathogenic somatic SNVs in *MTOR* were called even if their VAF was < 1% and/or with less than 10 reads for the alternative allele.

Variants identified by capture panel sequencing were classified as pathogenic if they were (1) somatic missense variants in mTOR-pathway activators already reported in FCD/HME or functionally proven to cause hyperactivation of the mTOR-pathway in vitro (2) or germline/somatic loss-of-function variants in mTOR-pathway repressors or in *SLC35A2*. All variants classified as pathogenic were further confirmed using alternative sequencing techniques: ultra-deep site-specific amplicon sequencing ( ≥ 9000X mean read depth) and droplet digital PCR (ddPCR) for low-VAF variants or conventional Sanger sequencing for germline or somatic variants with VAF > 10%. For ultra-deep site-specific amplicon sequencing, PCR amplicons (220–270 bp) specific for each variant were prepared following standard procedures and sequenced on a MiSeq sequencer (2 × 75 bp) at the iGenSeq sequencing facility of ICM. Bioinformatic analysis was conducted as reported above. We filtered for variants with base quality score ≥ 15 and mapping quality score ≥ 50. To exclude false-positive calls due to amplicon sequencing technical artifacts, we first estimated the probability value for true positive calls as recently described [20] and retained variants with a *p* value (*p*) < 0.01.

### Droplet digital PCR (ddPCR)

To validate recurrent somatic variants and assess the neural cell subtypes carrying the variants, we performed droplet digital PCR (ddPCR) (QX200 system, Bio-Rad Laboratories), a highly sensitive and specific technique based on sample partitioning following a Poisson distribution and allowing DNA absolute quantification. All reactions were prepared using the ddPCR Supermix for probes according to the manufacturer’s protocol. Specific ddPCR Mutation Detection Assays (FAM + HEX) were purchased from Bio-Rad to detect the *MTOR* variants p.Ser2215Phe and p.Thr1977Lys and the *PIK3CA* variant p.His1047Arg. Data were analyzed using the QX200 droplet reader and Quantasoft Analysis Pro software version 1.0.596. Validation of recurrent variants was performed using 50 ng of bulk brain DNA and blood DNA, leading to a statistically reliable quantification of the VAF in each sample. For the assessment of the neural cell subtypes carrying the variants, we performed ddPCR assays on pools of microdissected cells (see below), using the entire DNA extraction volume in each reaction; the number of cells isolated in each pool was dependent on the proportion of the different cell types in each tissue and tissue availability. For BCs and DNs, we amplified two independent pools for each patient: 15 and 22 BCs in FCD-56, 2 × 100 DNs in FCD-23, 2 × 20 DNs in FCD-24, 80 and 100 DNs in FCD-25, 2 × 25 DNs in FCD-56, and 20 and 50 DNs in HME-77. For normal appearing neurons (NNs), we tested: 1 × 100 NNs in FCD-23, 1 × 100 NNs in FCD-25, 1 × 100 NNs in HME-77, 1 × 55 NNs in FCD-24, 2 × 50 NNs in FCD-56. For glial cells (GCs), we tested: 1 × 105 GCs in FCD-23, 1 × 100 GCs in FCD-24, 1 × 100 GCs in HME-77, 1 × 200 GCs in FCD-25, and 2 × 50 GCs in FCD-56.

### Immunohistochemistry

Immunohistochemistry was performed on 20 μm cryosections with primary antibodies against Vimentin-V9 (1:200, Dako #M0725) to immunostain BCs and Smi-311 (1:250, BioLegend #837801) to immunostain DNs, and on 4 μm FFPE sections with a primary antibody against Ser240/244-phosphorylated ribosomal protein S6 (pS6^240/244^, 1:2000, Cell Signaling #5364), NeuN (1:500, Millipore #MAB377), and Olig2 (1:200, Epitomics) according to standard procedures, using an avidin–biotin peroxidase complex conjugation system (Vectastain ABC Elite; Vector Laboratories) and DAB as chromogen. Sections were counterstained with the standard hematoxylin and eosin (H&E) or hematoxylin alone and scanned in brightfield with a Nanozoomer scanner (Hamamatsu) at a 40X resolution. Images were captured with the software NDP.view2.

### Laser-capture microdissection and ddPCR

Laser-capture microdissection (LCM) was performed using a Leica LMD7000 system on 20 μm frozen brain sections mounted on PEN-membrane slides after rapid cresyl-violet and eosin (CV&E) staining, adapted from a previously reported protocol [36]. Cell subpopulations were selected as follows: (1) BCs: large (mean longest cell body diameter *d* = 39 μm), ovoid-shaped cell body, often presenting an eccentric nucleus or multiple nuclei, and glassy eosinophilic cytoplasm without Nissl substance; (2) DNs: cytomegalic neurons (*d* > 20 μm), with Nissl substance cytoplasmic aggregates and abnormal soma shape; (3) morphologically normal appearing neurons (NNs): oval shape, without Nissl substance cytoplasmic aggregates and *d* = 10–15 μm; and (4) glial cells (GCs): small round-shaped cells (*d* = 5–7 μm). The microdissected cells were collected in AdhesiveCap 500 Opaque tubes (Zeiss) for DNA extraction as previously described [11].

For the assessment of the cell types carrying the pathogenic variants, we isolated BCs, DNs, NNs, and GCs in a subset of patients selected according to the histological quality of the frozen sections (2–6 sections/patient) and the number of BCs and DNs: one FCD2b case (FCD-56), three FCD2a cases (FCD-23, FCD-24 and FCD-25), and one HME case (HME-77).

To confirm the loss-of-heterozygosity (LOH) in patient FCD-36, we isolated pools of BCs, DNs, NNs, and GCs from two adjacent brain sections.

### Balloon cells (BCs) counting

To calculate the density of BCs in selected samples (five panel-negative and four panel-positive FCD2b cases), we performed H&E staining on three 20 μm frozen brain section adjacent to the sections used for DNA extraction, scanned the slides with a Nanozoomer scanner (Hamamatsu) at a 40X resolution, captured the images with the software NDP.view2, and counted the number of BCs present in each entire section. For every patient analyzed, we subsequently calculated the BCs density mean by dividing the total BCs count by the total area of the section.

### Statistical analyses

Genotype–phenotype correlations were conducted using Fisher’s exact test to compare categorical data, Mann–Whitney test to compare data distribution in independent groups and Kruskal–Wallis test for multiple comparisons. Two-tailed *p* values *p* < 0.05 were considered significant. Statistical analyses were performed with Prism 8 (GraphPad Software).

## Results

### Overall genetic findings

**Fig. 1:**
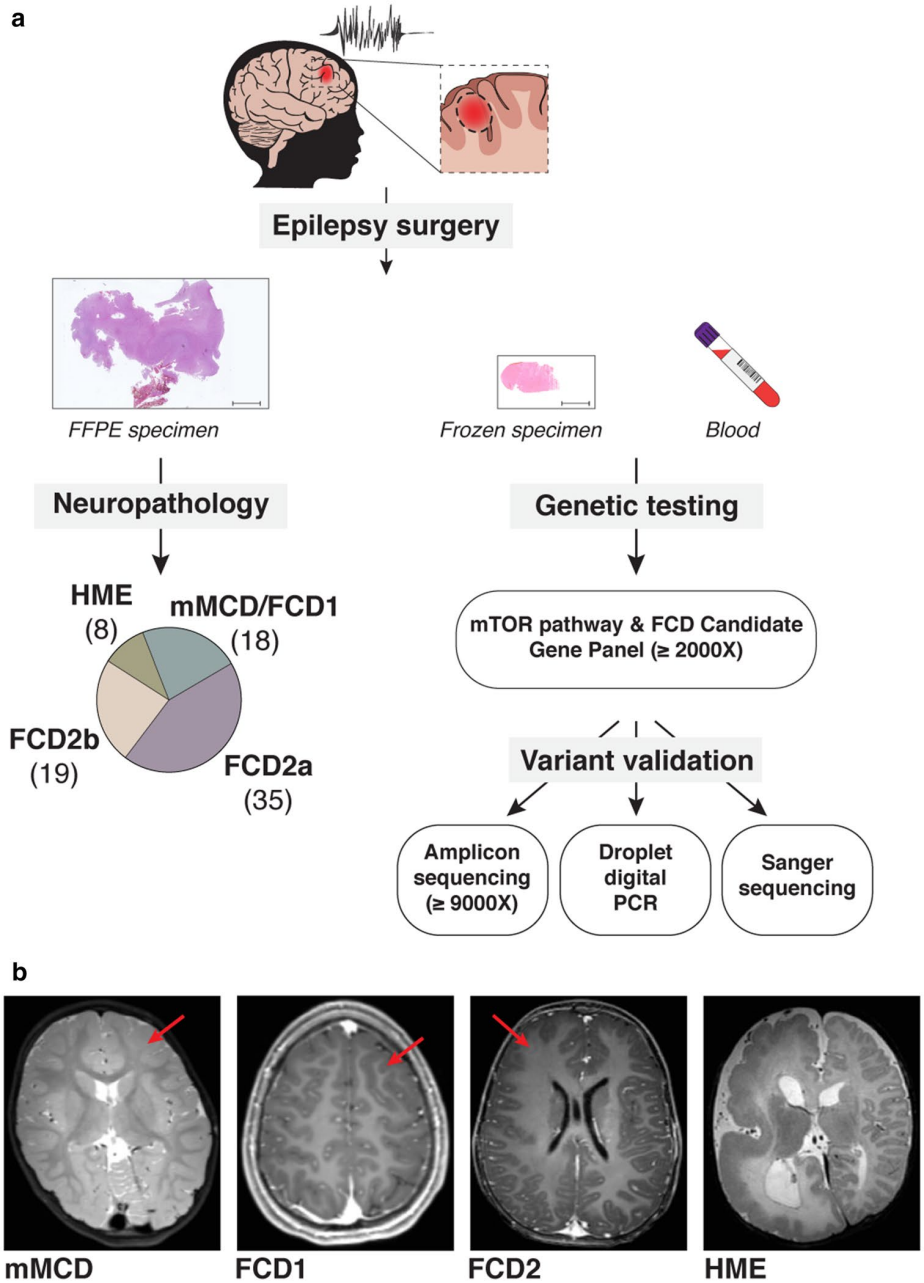
Study workflow. **a** The study includes children who underwent epilepsy surgery with suspected cortical brain malformation of development, including mMCD, FCD (types 1 and 2), or HME. For each patient, we collected both FFPE (formalin-fixed paraffin-embedded tissue) and fresh frozen brain specimens, and matched blood samples. Neuropathological examination allowed classification of mild MCD (mMCD), FCD1, FCD2a, FCD2b, and HME subtypes in 80 samples. Targeted capture-based panel sequencing of mTOR pathway and FCD candidate genes revealed germline, somatic, and two-hits variants, which were subsequently confirmed by alternative sequencing methods (amplicon or conventional Sanger) or droplet digital PCR. **b** Representative 3 T MRI images are shown for mMCD (FCD-1 case), FCD1 (FCD-9 case), FCD2 (FCD-30 case) and for HME (HME-76 case)

**Fig. 2:**
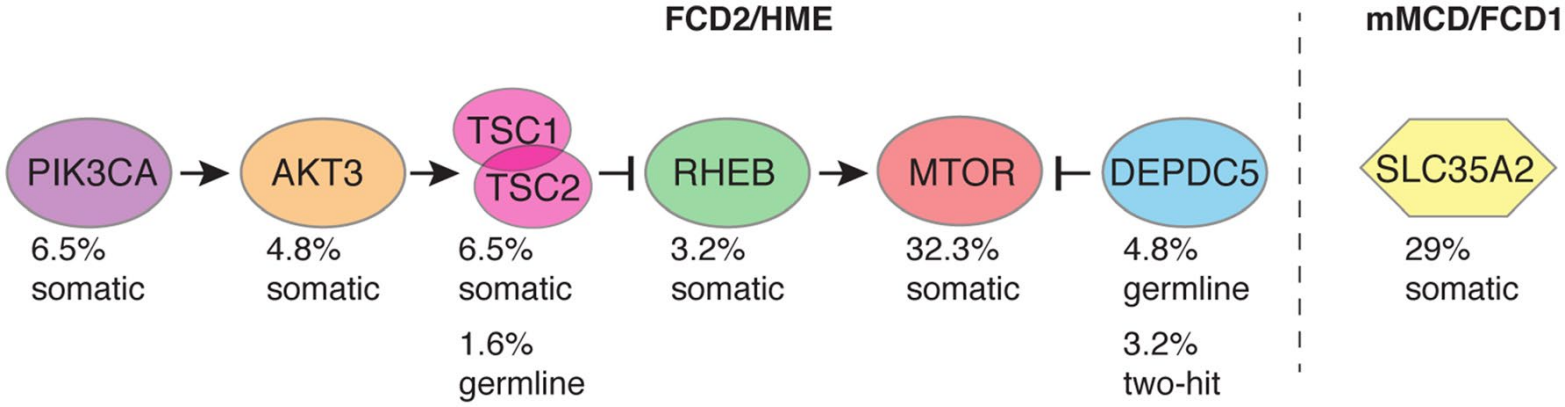
Overall genetics findings. Global diagnostic yield among mMCD/FCD1, FCD2, and HME cases are illustrated. The percentage of patients carrying pathogenic variants among the panel-positive cases (*n* = 43) is indicated to denote the contribution of each gene to the pathogenesis of mMCD/FCD/HME

We designed a targeted sequencing and bioinformatic pipeline for the detection of somatic single nucleotide variants (SNVs) or indels with low-variant allele frequency (VAF), to comprehensively assess the genetic etiology of distinct MCD subtypes (i.e., mMCD, FCD1, FCD2, and HME) in a cohort of 80 surgical cases with cortical malformations (the study workflow is illustrated in Fig. 1). We performed targeted capture deep sequencing ( ≥ 2000X mean read depth) of 25–30 mTOR-pathway genes and other FCD candidate genes (among which *SLC35A2*) on matched blood–brain DNA samples. All together, we identified germline, somatic, and somatic two-hit variants in 29% of mMCD/FCD1 cases (*SLC35A2* gene), 59% (32/54) of FCD2 cases, and 87.5% (7/8) of HME cases in genes of the mTOR pathway (*AKT3*, *DEPDC5*, *MTOR*, *PIK3CA, RHEB, TSC1, TSC2*) (Table 1, Fig. 2).

**Table 1:** Summary of genetic findings

Patient ID	Neuropathology	Gene/cDNA/protein variant	VAF (%) in brain/blood (gene panel)	VAF (%) in brain/blood (replication)
FCD-19	FCD2a	MTOR: c.4376C>A/p.Ala1459Asp^a^	0.45/0	Amplicon: 0.89/NA
FCD-54	FCD2b	MTOR: c.4376C>A/p.Ala1459Asp^a^	1.3–1.6/0	Amplicon: 1.16–1.68/NA
HME-73	HME/FCD2b	MTOR: c.4376C>A/p.Ala1459Asp^a^	9.2/0	Amplicon: 10.49/NA
FCD-55	FCD2b	MTOR: c.4379 T>C/p.Leu1460Pro^a^	3.4/0	Amplicon: 3.5/NA
FCD-20	FCD2a (large)	MTOR: c.4379 T>C/p.Leu1460Pro^a^	18.6/0	Amplicon: 15.67/NA
FCD-56	FCD2b	MTOR: c.5930C>A/p.Thr1977Lys^a^	5.5/0	Amplicon: 4.67/0; ddPCR: 3.67/0
FCD-21	FCD2a	MTOR: c.6644C>T/p.Ser2215Phe^a^	0.89/0	ddPCR: 0.59–5.74/0
FCD-22	FCD2a	MTOR: c.6644C>T/p.Ser2215Phe^a^	1.2/0	ddPCR: 1.07/0
FCD-57	FCD2b	MTOR: c.6644C>T/p.Ser2215Phe^a^	1.28/0	ddPCR: 1.25/0; 0.4 in the perilesional tissue
FCD-58	FCD2b	MTOR: c.6644C>T/p.Ser2215Phe^a^	1.5/0	ddPCR: 0.742/0
FCD-23	FCD2a	MTOR: c.6644C>T/p.Ser2215Phe^a^	1.5–2.7/0	ddPCR: 1.96–5/0
FCD-24	FCD2a	MTOR: c.6644C>T/p.Ser2215Phe^a^	2.1/0	ddPCR: 2.32/0
FCD-59	FCD2b	MTOR: c.6644C>T/p.Ser2215Phe^a^	3.4/0	ddPCR: 3.7/0
FCD-60	FCD2b	MTOR: c.6644C>T/p.Ser2215Phe^a^	6.5/0	ddPCR: 8.43/0
FCD-25	FCD2a (hemispheric)	MTOR: c.6644C>T/p.Ser2215Phe^a^	8.54/NA	ddPCR: 8.73/NA
FCD-26	FCD2a	MTOR: c.6644C>A/p.Ser2215Tyr^a^	0.37/0	Amplicon: 0.25/NA
FCD-27	FCD2a	MTOR: c.6644C>A/p.Ser2215Tyr^a^	0.42/0	Amplicon: 0.44/NA
FCD-28	FCD2a	MTOR: c.6644C>A/p.Ser2215Tyr^a^	0.57/NA	NA
FCD-61	FCD2b	MTOR: c.6644C>A/p.Ser2215Tyr^a^	3.7/0	Amplicon: 4.52/NA, 1.5 in the perilesional tissue
FCD-29	FCD2a	MTOR: c.7498A>T/p.Ile2500Phe^a^	2.3/0	Amplicon: 1.77%/NA
FCD-30	FCD2a	AKT3**:** c.49G>A/p.Glu17Lys^a^	1/0	Amplicon: 1.1/NA
FCD-31	FCD 2a (hemispheric)	AKT3**:** c.49G>A/p.Glu17Lys^a^	2–3/0	Amplicon: 2.1–2.3/NA
HME-74	HME/FCD2a	AKT3**:** c.49G>A/p.Glu17Lys^a^	11.9/0	Amplicon: 11.6/NA
HME-75	HME/FCD2a	PIK3CA: c.1624G>A/p.Glu542Lys^a^	7.5/0	Amplicon: 7.04/-
HME-76	HME/FCD2a	PIK3CA: c.1624G>A/p.Glu542Lys^a^	33.5/0	Sanger: confirmed/NA
HME-77	HME/FCD2a	PIK3CA: c.3140A>G/p.His1047Arg^a^	21.2/0	ddPCR: 21.7/0
HME-78	HME/FCD2a	PIK3CA: c.3140A>G/p.His1047Arg^a^	29.8/0	ddPCR: 15.8/0
FCD-62	FCD2b	RHEB**:** c.119A >T/p.Glu40Val^a^	8.9/0	Amplicon: 8.4–8.8/NA
HME-79	HME/FCD2b	RHEB**:** c.[105C>A,104A > T]/p.Tyr35Leu	17.6/0	Sanger: confirmed/NA
FCD-63	FCD2b	TSC1**:** c.1525C >T/p.Arg509*^a^	4.6/2	Amplicon: 3.4–4.2/2.7
FCD-64	FCD2b	TSC1**:** c.1907_1908delAG/p.Glu636fs*51^a^	3.7/2.2	Amplicon: 4.12/1.01
FCD-65	FCD2b	TSC2**:** c.5228G >A/p.Arg1743Gln^a^	1/0	Amplicon: 1.4/NA
FCD-66	FCD2b	TSC2**:** c.2380C>T/p.Gln794*	1.5/0	Amplicon: 2.15/NA
FCD-32	FCD2a	TSC2**:** c.3725dupA/p.Glu1243fs	51/49	Sanger: confirmed germline
FCD-33	FCD2a	DEPDC5**:** c.856C >T/ p.Arg286*;c.865C>T/p.Gln289*	49/10; 52/0	Sanger and Amplicon: confirmed germline; 10/0, 0.3 in the perilesional tissue
FCD-34	FCD2a	DEPDC5**:** c.279 + 1G >A	49/47	Sanger: confirmed germline
FCD-35	FCD2a	DEPDC5**:** c.715C>T/p.Arg239*	51/49	Sanger: confirmed germline
FCD-36	FCD2a (hemispheric)	DEPDC5**:** c.3021 + 1G >A	65/48	Sanger: confirmed germline + somatic LOH
FCD-37	FCD2a	DEPDC5**:** c.4151_4152insC/p.Glu1385fs	53/49	Sanger: confirmed germline
FCD-1	mMCD2	SLC35A2**:** c.801C >G/p.Tyr267*	12.1/0	Sanger: confirmed/NA
FCD-2	mMCD2	SLC35A2**:** c.634_635delTC/p.Ser212Leufs*9	22.6/0	Sanger: confirmed/NA
FCD-3	mMCD2 (bilobar)	SLC35A2**:** c.886_888delCTC/p.Leu296del	22.4/0	Amplicon: 24.02/NA
FCD-4	mMCD2	SLC35A2**:** c.804dupA/p.Pro269Thrfs*24	32.7/0	Sanger: confirmed/NA

Subjects FCD-22–23, FCD-59–61, and FCD-33–37 have been previously reported [3, 32, 41]. When multiple brain specimens were sequenced, a range of the obtained variant allele frequencies (VAFs) is reported. Perilesional tissue: brain tissue surrounding the seizure-onset zone. ddPCR: droplet digital PCR

*NA* not available

^a^Variant reported in COSMIC database

### Clinical features of the cohort

We enrolled 80 sporadic children (43 males and 37 females) with refractory focal epilepsy who underwent resective neurosurgery and had a neuropathological diagnosis of mMCD, FCD, or HME. The cohort comprised 18 mMCD/FCD1 (with microcolumns reported in two cases classified as FCD1a and abnormal tangential cortical lamination in two cases classified as FCD1b), 35 FCD2a, 19 FCD2b, and eight HME cases (with FCD2a/2b histopathology) according to the current ILAE classification [7]. Clinico-neuropathological findings are summarized in Table 2 and extensively described in Supplementary Table 1 (Online Resource 2).

**Table 2:** Clinical data summary of panel-positive cases

	Entire cohort (80 cases)	MTOR (20 cases)	AKT3 (3 cases)	PIK3CA (4 cases)	RHEB (2 cases)	TSC1/2 somatic (4 cases)	DEPDC5/TSC Germline (6 cases)	SLC35A2 (4 cases)
Demographic data								
Mean age at seizure onset (range)	1.7 y (1 d–13 y)	1.2 y (1 d–10 y)	2 m (1 d–6 m)	10 d (2 d–1 m)	3 m (3d–6 m)	1.5 y (1.2–2 y)	1.9 y (1 d–6 y)	1.25 y (5 m–2.5 y)
Mean duration of epilepsy (range)	4.7 y (2 m–15 y)	5.9 y (3 m–15 y)	2 y (4 m–4 y)	7 m (2 m–1 y)	11 m (5 m–1.5y)	2 y (1 y–2.7 y)	7.4 y (3 m–12.8 y)	4 y (1.5 y–5.6 y)
Seizure frequency: daily	69 (86.25%)	17 (85%)	3 (100%)	4 (100%)	1 (50%)	4 (100%)	3 (50%)	4 (100%)
Seizure frequency: weekly	11 (13.75%)	3 (15%)	0	0	1 (50%)	0	3 (50%)	0
History of focal seizures exclusively	61 (76.25%)	16 (80%)	3 (100%)	2 (50%)	2 (100%)	3 (75%)	6 (100%)	0
History of infantile spasm exclusively	11 (13.75%)	3 (15%)	0	0	0	1 (25%)	0	4 (100%)
History of focal seizures and infantile spasm	8 (10%)	1 (5%)	0	2 (50%)	0	0	0	0
Sleep and wake seizures	57 (71.25%)	14 (70%)	1 (33.3%)	3 (75%)	1 (50%)	2 (50%)	4 (66.7%)	3 (75%)
Sleep seizures only	17 (21.25%)	5 (25%)	1 (33.3%)	0	0	2 (50%)	2 (33.3%)	0
Normal 3 T MRI	4 (5%)	0	0	0	0	0	0	0
PET hypometabolism	30/31 (97%)	7/7 (100%)	N/A	N/A	N/A	1/1 (100%)	4/4 (100%)	1/1 (100%)
Normal neurological examination	54 (67.5%)	11 (55%)	1 (33.3%)	0	0	4 (100%)	5 (83.3%)	4 (100%)
Normal neuropsychological examination	30 (37.5%)	6 (30%)	0	0	0	3 (75%)	5 (83.3%)	0
Mild developmental delay	29 (36.25%)	10 (50%)	1 (33.3%)	1 (25%)	0	1 (25%)	0	2 (50%)
Moderate developmental delay	15 (18.75%)	3 (15%)	2 (66.7%)	3 (75%)	1 (50%)	0	1 (16.7%)	1 (25%)
Severe developmental delay	6 (7.5%)	1 (5%)	0	0	1 (50%)	0	0	1 (25%)
Epilepsy surgery								
Mean age at surgery (range)	6.4 y (3 m–16.7 y)	7.2 y (3 m–16.1 y)	2.1 y (4 m–1.3)	8 m (3.6 m–1.1 y)	1.2 y (5 m–2 y)	3.4 y (2.4 y–4.8 y)	9.3 y (3 m–15.1 y)	5.2 y (3 y–8 y)
Mean follow-up duration (range)	2.5 y (7.3 m–3.9 y)	2.5 y (8.4 m–3.9 y)	2.7 y (2 y–3.9 y)	2.2 y (1.4 y–3.2 y)	2.6 y (1.8 y–3.4 y)	2.8 y (1.1 y–3.5 y)	2.3 y (1.1 y–3.9 y)	2.6 y (1.3 y–3.9 y)
Multiple surgeries	26 (32.5%)	9 (45%)	2 (66.7%)	0	1 (50%)	0	2 (33.3%)	2 (50%)
Frontal resection	30 (37.5%)	5 (25%)	1 (33.3%)	0	0	3 (75%)	3 (50%)	2 (50%)
Parietal resection	6 (7.5%)	3 (15%)	0 (0%)	0	0	0	0	0
Temporal resection	9 (11.25%)	1 (5%)	0 (0%)	0	0	0	0	0
Monolobar resection	50 (62.5%)	9 (45%)	1 (33.3%)	0	0	3 (75%)	0	2 (50%)
Multilobar resections	17 (21.25%)	9 (45%)	0	0	0	1 (25%)	1 (16.7%)	2 (50%)
Hemispherotomy	13 (16.25%)	21 (10%)	2 (66.7%)	4 (100%)	2 (100%)	0	1 (16.7%)	0
Surgical outcome Engel class 1	55 (68.75%)	14 (70%)	2 (66.7%)	4 (100%)	2 (100%)	4 (100%)	3 (50%)	3 (75%)
Surgical outcome Engel class 2	6 (7.5%)	0	0	0	0	0	1 (16.7%)	0
Surgical outcome Engel class 3	14 (17.5%)	6 (30%)	0	0	0	0	2 (33.3%)	0
Surgical outcome Engel class 4	5 (6.25%)	0	1 (33.3%)	0	0	0	0	1 (25%)
Neuropathology								
mMCD2	11 (13.75%)	0	0	0	0	0	0	4 (100%)
FCD1	7 (8.75%)	0	0	0	0	0	0	0
FCD2a	35 (43.75%)	11 (55%)	2 (66.7%)	0	0	0	6 (100%)	0
FCD2b	19 (23.75%)	8 (40%)	0	0	1 (50%)	4 (100%)	0	0
HME/FCD2	8 (10%)	1 (5%)	1 (33.3%)	4 (100%)	1 (50%)	0	0	0

Demographic clinical data for the full cohort or stratified along with the nature of the mutated gene (numbers of patients in each group is indicated). For patients who underwent multiple surgeries, clinical data and histopathological diagnoses from the last surgical procedure are indicated. d: day; m: month; y: years. No hippocampal sclerosis was observed in patients with temporal lobe cortical dysplasia. No interictal hypsarrhythmia EEG pattern was observed among patients with infantile spasms. Developmental delay has been classified according to the standard definition: mild IQ = 50–70, moderate IQ = 30–50, severe IQ < 30. Neuropsychological evaluation was performed in 76 patients

Mean age at seizure onset was 1.6 ± 2.7 years (from 0 to 13 years) and mean epilepsy duration before surgery was 4.4 ± 4.0 years (8.4 months in HME and 5.2 years in mMCD/FCD). All patients had focal seizures, 23% of which presented as focal infantile spasms characterized by stereo-EEG (SEEG) bursts of rapid rhythms. Topography of the seizure-onset zone (SOZ) was assessed by scalp video electroencephalography (EEG) and/or invasive intracranial SEEG. Among patients investigated by SEEG, 64% (37/58) had a monolobar SOZ, predominantly located in the frontal lobe (54%). Magnetic resonance imaging (MRI, 3 Tesla) revealed a dysplasia in 79% of the cases, while only a blurring of the grey–white matter junction was observed in 8.8% of the patients, and four patients (5%) had normal MRI. We observed a higher occurrence of moderate-to-severe developmental delay among patients who underwent a hemispherotomy, compared to those who underwent focal resections (10/13 versus 11/67, *p* < 0.0001). Autistic features were reported in 24% (19/80) of the children with or without cognitive delay. Mean age at surgery was 6.4 ± 4.9 years (8.9 ± 4.8 months for HME, 7 ± 4.7 years for mMCD/FCD). Monolobar resections were performed in 50 patients, multilobar resections in 17 patients, and hemispherotomies in 13 patients. Mean postsurgical follow-up duration was 2.5 ± 1.0 years (from 7.3 months to 3.9 years). The post-operative outcome was evaluated using the Engel Epilepsy Surgery Outcome Scale: 68.75% of the patients were Engel class 1 (free of seizures), 7.5% were Engel class 2 (rare seizures), and 23.75% were Engel class 3 or 4 (worthwhile improvement or no worthwhile improvement, respectively). Seizure control was achieved after multiple surgeries in 19 patients (from two to five surgical interventions, see Supplementary Table 1, Online Resource 2). The poor surgical outcome in the remaining cases may have result from incomplete resection.

**Fig. 3:**
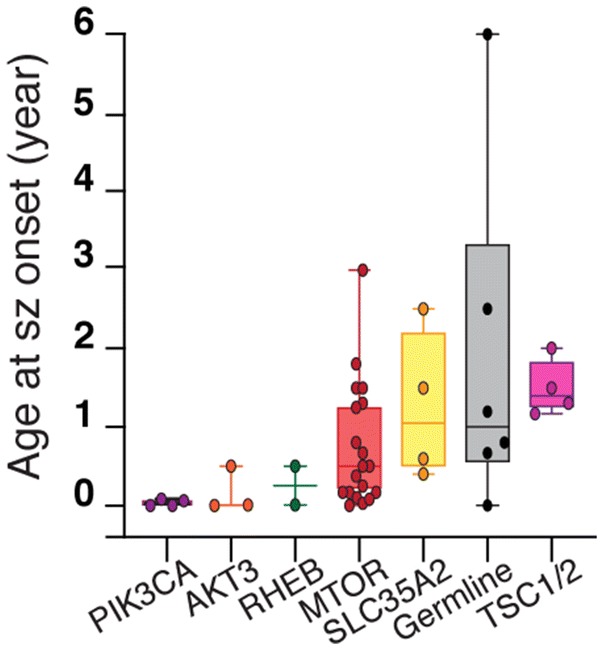
Genotype–phenotype correlations. Comparison of age at seizure (sz) onset between groups of patients with variants in different genes, reported as boxplots with minimum, maximum, average, and standard deviation (Kruskal–Wallis test with multiple comparisons). Germline group includes five *DEPDC5* germline cases and one *TSC2* germline case. TSC1/2 group includes only somatic variants

We then asked whether the presence of a given brain somatic variant was correlated with specific clinical features. A significant difference in the distribution of the age at seizure onset was observed among groups of patients, dependent on the genetic etiology. Indeed, age at onset was earlier in patients with either *PIK3CA* (neonatal period), *AKT3,* or *RHEB* (before 6 months of age) variants, together with a poor cognitive outcome, compared to patients with somatic variants in *MTOR*, *TSC1/2,* and *SLC35A2* or germline variants in *TSC2* or *DEPDC5*, presenting a greater variability (*p* = 0.039, Fig. 3). This is also consistent with higher brain mosaicism rates (from 7.5 to 34%) of variants found in *PIK3CA*, *AKT3* and *RHEB*, reflecting an earlier timing of variant occurrence.

A higher occurrence of infantile spasms was observed in patients with *SLC35A2* somatic variants (4/4 subjects) compared to panel-negative FCD1/mMCD cases (3/14, *p* = 0.011). A smaller fraction of panel-positive FCD2/HME cases (7/34) with somatic variants in *MTOR*, *PIK3CA,* or *TSC2* also presented infantile spasms.

All panel-positive cases presented MRI abnormalities, including all four *SLC35A2*-mutated patients: three with a frontal FCD, one with a temporo-occipital FCD. A normal 3 T MRI was observed in only four cases of the entire cohort, all panel-negative and with a good surgical outcome (Engel class 1 at > 2 years).

We also asked whether the detection of a variant in a given gene can predict the post-operative outcome. We thus compared all subjects divided into two groups with either a good surgery outcome (Engel class 1/2) or a poor surgery outcome (Engel class 3/4). Overall, 76% (61/80) of the patients had a good surgery outcome (Engel class 1/2). Similar proportions were found in different gene subgroups: 75% of *SLC35A2* cases, 70% of *MTOR* cases and 67% of patients with germline variants in *TSC1/2* or *DEPDC5* were Engel class 1/2. We, therefore, did not observe any predictive correlation between a given mutated gene and surgery outcome.

### Neuropathological and genetic findings in mMCD/FCD1

**Fig. 4:**
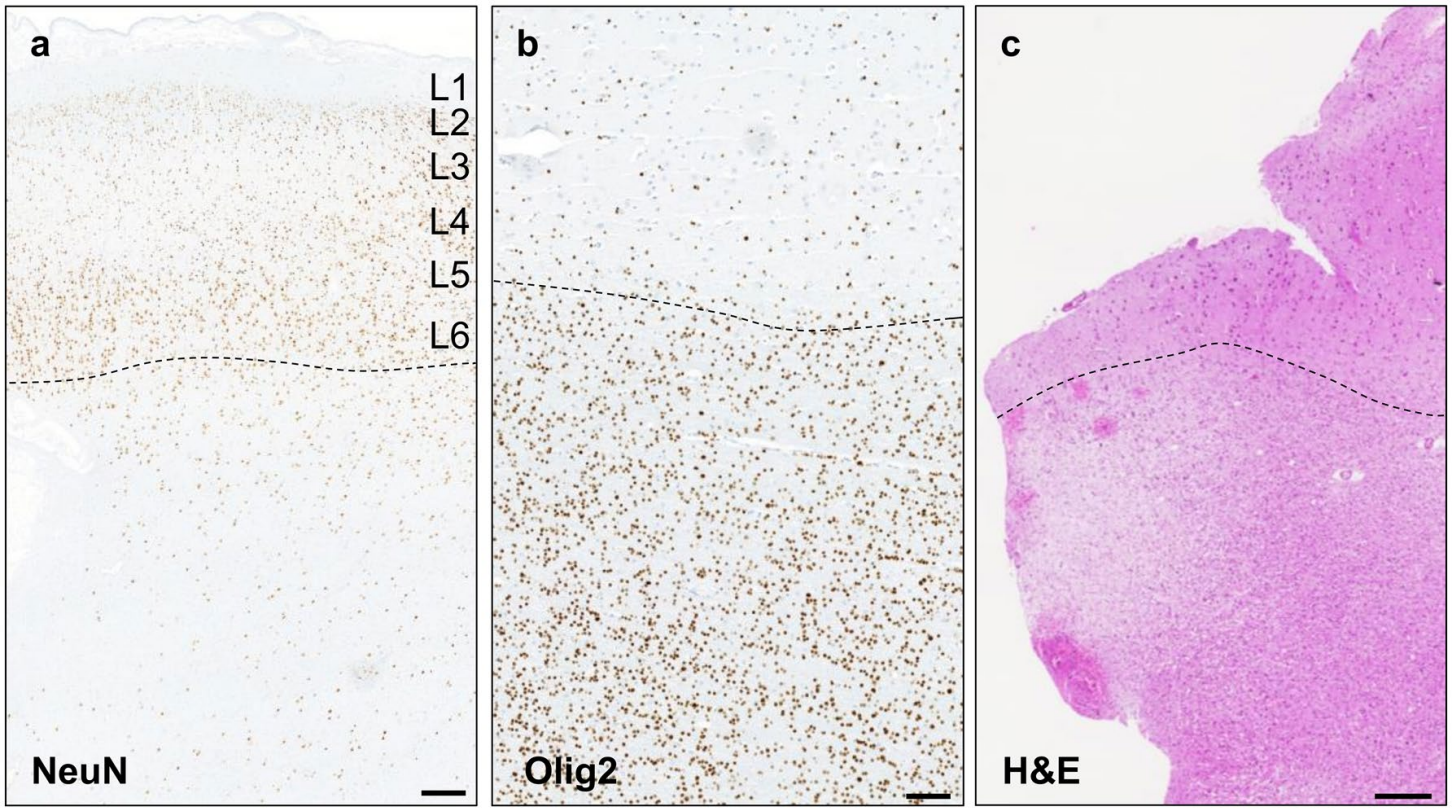
Histological findings in *SLC35A2*-mMCD2 brain specimens. **a** NeuN immunoreactivity of the middle frontal cortex region from patient FCD-2 shows blurred grey–white matter border (dashed line) and excess of heterotopic neurons ( > 30/mm^2^); scale bar: 1 mm. **b** Increased oligodendrocyte density in the white matter (below the dashed line) in the fronto-pre-central brain tissue from patient FCD-4; scale bar: 500 µm. **c** H&E staining of the pre-motor cortex tissue in patient FCD-4 shows white matter pallor; scale bar: 250 µm

We identified brain-only loss-of-function *SLC35A2* variants in four male mMCD2 cases. The gene was screened in 14 cases out of 18, representing a genetic burden of 29% (4/14) in mMCD/FCD1 cases. All mutated cases displayed a blurred grey–white matter border with an excess of heterotopic neurons in the deep white matter ( > 30 neurons/mm^2^ at > 500 µm from the grey–white matter border), while the cortical lamination appeared mostly conserved (Fig. 4a). An increased oligodendrocyte density (Fig. 4b) and white matter pallor (Fig. 4c) were also observed.

*SLC35A2* variant allele frequencies (VAFs) ranged from 12 to 43%. All variants were classified as pathogenic according to ACMG guidelines for variant classification [42]: p.Tyr267*, p.Ser212Leufs*9, p.Pro269Thrfs*24 fulfilled criteria PVS1, PS2, PM2, PM4, and PM6, while the variant p.Leu296del fulfilled criteria PS2, PM2, PM4, and PM6. All variants were in fact absent from gnomAD database, occurred de novo during neurodevelopment, and were loss-of-function variants in a haploinsufficient gene previously associated with a mendelian disorder.

### Neuropathological and genetic findings in FCD2/HME

#### Variants in mTOR activators

In FCD2/HME, we identified pathogenic variants exclusively in genes belonging to the mTOR pathway. Notably, 47% (29/62) of the cases presented brain-only gain-of-function missense variants in activators of the pathway (*AKT3*, *MTOR*, *PIK3CA,* and *RHEB*). VAFs ranged from as low as 0.37% up to 8.9% among FCD2, and up to 18.6% in one large FCD2a. In HME, VAFs ranged from 7.5% up to 34%. Single nucleotide variants (SNVs) in *MTOR* gene were the most frequent, accounting for 64.5% (20/31) of the cases (19 FCD2 cases, 1 HME case): all variants except p.Ile2500Phe were recurrent SNVs and located in mutational hotspots of the FAT and kinase domains of mTOR protein. *PIK3CA* variants were exclusively found in HME cases. A variant gradient with a higher mosaic rate in the seizure-onset zone compared to the surrounding epileptogenic zone was observed in three cases with multiple brain specimens available (FCD-33, FCD-57, and FCD-61, Table 1).

#### Variants in mTOR repressors

We identified loss-of-function variants in negative regulators of mTOR pathway in ten FCD2 cases. Four FCD2b cases had a somatic variant in *TSC1*/*TSC2* genes; one FCD2a case had a germline pathogenic variant in *TSC2* and was subsequently diagnosed with a mild form of tuberous sclerosis complex. Five FCD2a patients had *DEPDC5* germline loss-of-function variants. For mTOR repressors, a two-hit mutational mechanism is assumed to cause the focal dysplastic lesion [5, 10, 31, 41]. In this cohort, a somatic second-hit SNV was detected in one *DEPDC5* case (FCD-33) [41]. Moreover, in one patient with a germline splice-site SNV in *DEPDC5* (FCD-36 with a hemispheric FCD2a), we suspected a somatic loss-of-heterozygosity (LOH) due to biased VAF of the germline variant in the brain and blood samples (65% allele frequency in the brain and 48% in the blood, see below).

#### Activation of mTOR pathway in FCD2 specimens

**Fig. 5:**
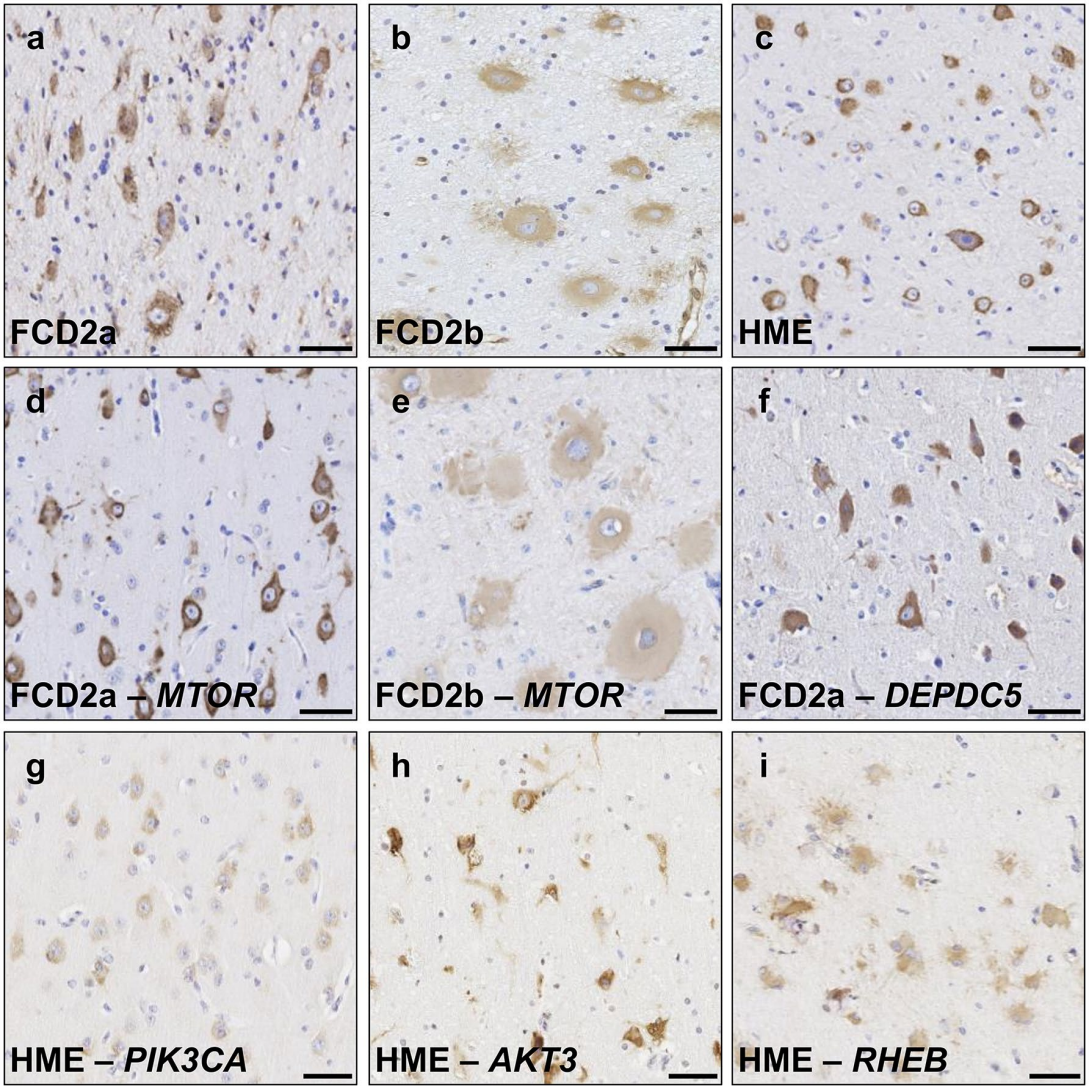
Activation of the mTOR pathway in FCD2/HME cases. pS6 (pS6 ^Ser240/244^) immunostaining on 4 μm-FFPE brain sections in panel-negative (**a**–**c**) and panel-positive FCD2/HME cases (**d**–**i**). Strong pS6 immunoreactivity in DNs and BCs was detected in all cases, though signals appeared less intense in BCs. Panel-negative representative cases are shown to illustrate: FCD2a in **a** (FCD-40), FCD2b in **b** (FCD-68) and HME in **c** (HME-80). Panel-positive representative cases: FCD2a *MTOR*:p.Ser2215Phe in **d** (FCD-24), FCD2b *MTOR*:p.Thr1977Lys in **e** (FCD-56), FCD2a *DEPDC5*:p.Arg239* in **f** (FCD-35). HME cases with variants in different genes are reported in panels **g–i**: *PIK3CA*:p.Glu542Lys in **g** (HME-75), *AKT3*:p.Glu17Lys in **h** (HME-74); *RHEB*:Tyr35Leu in **i** (HME-79). Scale bar: 50 µm

We next investigated the activation of the mTOR-signaling cascade in FCD2/HME panel-positive and panel-negative specimens (i.e., with or without an identified mTOR-pathway variant). In all FCD2 cases (*n* = 25 analyzed cases), we detected strong phosphorylation of the downstream effector ribosomal protein S6 (pS6), a standard readout of the mTOR activity, in DNs and BCs (Fig. 5 and Supplementary Fig. 1, Online Resource 1). We also compared the pS6-immunostaining profile among cases with variants occurring in different genes. We did not observe major differences among cases with distinct variants in *MTOR* (Fig. 5d, e) or with *AKT3*, *RHEB,* or *DEPDC5* variants (Fig. 5f, h, i). A less intense pS6 staining was, however, consistently observed in brain specimens from *PIK3CA-*HME subjects, reflecting possible complex feedback regulatory mechanisms occurring upon PIK3CA hyperactivity (Fig. 5g). These observations provide evidence that all FCD2 cases presenting a mosaic pattern of mTOR hyperactivation are likely to be linked to somatic variants in genes belonging to the mTOR cascade.

#### Linking neuropathology to genetics: balloon cells and dysmorphic neurons carry pathogenic variants

Subsequently, we aimed to search for the somatic variants in specific neural cell subtypes. Since hyperactivation of mTOR pathway is assumed to account for the enlarged cell phenotype of BCs and DNs, we postulated that these pathological cells are the carriers of the pathogenic variants.

**Fig. 6:**
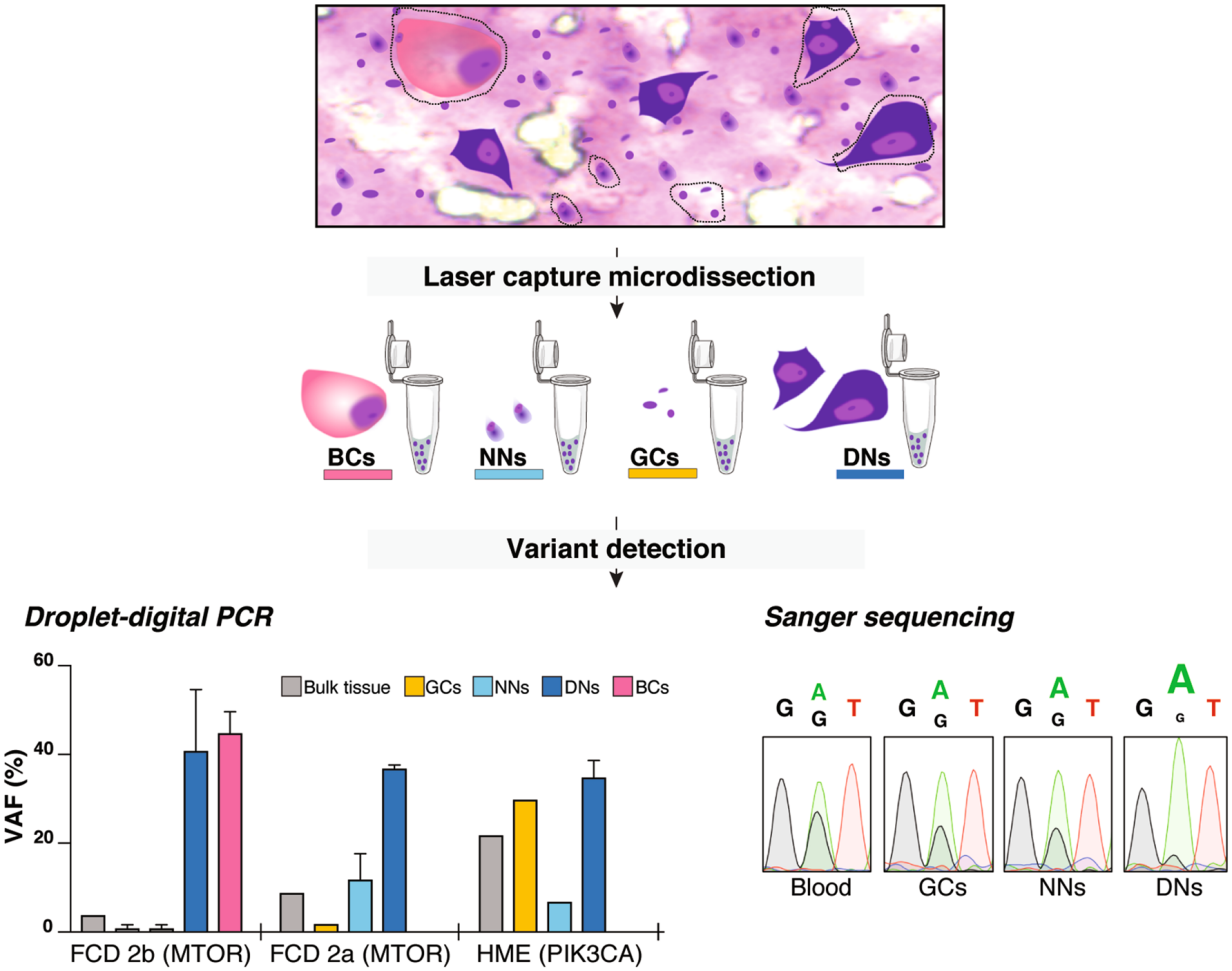
Balloon cells and dysmorphic neurons carry pathogenic variants. Characterization of variant-carrying cells and LOH evidence by laser-capture microdissection. DNs, BCs, neurons with normal appearance (NNs) and glial cells (GCs) were laser-captured and pooled for DNA extraction. Left panel: For *MTOR* and *PIK3CA* variants detection, we performed droplet digital PCR on DNA extracted from various pools: 20–100 cells for DNs, two pools of 15 and 22 cells for BCs, pools of 50–100 cells for NNs and pools of 100–200 cells for GCs. The histogram illustrates the variant allele frequency (VAF, in percentage) detected by droplet digital PCR (ddPCR) in three patients (FCD-25, FCD-56 and HME-77). A single ddPCR experiment was performed on bulk DNAs and for laser-captured NNs and GCs in FCD2a and HME samples. For all laser-captured cell types in FCD2b sample and for laser-captured DNs in all samples, the ddPCR experiment was performed in duplicate to achieve significance: mean variant allele frequency values and standard error of the means are reported in these cases. Right panel: Sanger sequencing on DNA extracted from pools of 250 DNs, 300 NNs and 300 GCs was performed to confirm *DEPDC5* somatic LOH in patient FCD-36

To test this hypothesis, we isolated DNs, BCs, morphologically normal neurons and glial cells by LCM, based on their morphology on cresyl-violet/eosin stained frozen brain sections from one FCD2a case with *MTOR*:p.Ser2215Phe variant (FCD-25), one FCD2b case with *MTOR*:p.Thr1977Lys variant (FCD-56), and one HME case with *PIK3CA*:p.His1047Arg variant (HME-77). The presence of vimentin-positive BCs and Smi-311-positive DNs was confirmed on frozen sections adjacent to those used for LCM (data not shown). We discovered a strong VAF enrichment (up to 45%) in both pools of DNs and BCs compared to morphologically normal neurons (down to 1.4%) and glial cells (down to 1.4%) or bulk tissue (Fig. 6). This finding indicates that > 90% of DNs and BCs indeed carry the pathogenic variants. We further confirmed these results in two additional FCD2a cases with *MTOR*:p.Ser2215Phe variant (Supplementary Table 2, Online Resource 1). In the *PIK3CA*:p.His1047Arg HME case, we found an enrichment of the somatic variant in glial cells ( ~ 60% of cells), probably reflecting an early mutational event in common neuroglial progenitors. Together these results show that both DNs and BCs are the main carriers of the mTOR hyperactivating variants in FCD2 cases.

Finally, we took advantage of the strong enrichment of somatic variants in DNs and BCs to validate the hypothesis of a somatic second-hit *DEPDC5* LOH in patient FCD-36 (see above section). We performed Sanger sequencing on DNA extracted from pools of LCM-isolated neural cells. While we clearly detected both wild type (WT) and mutant alleles in pools of 300 glial cells and 300 morphologically normal neurons, the WT allele was nearly undetectable in a pool of 250 DNs indicating a mosaic loss of the WT allele in these cells (Fig. 6).

#### Balloon cells density correlates with genetic findings

**Fig. 7:**
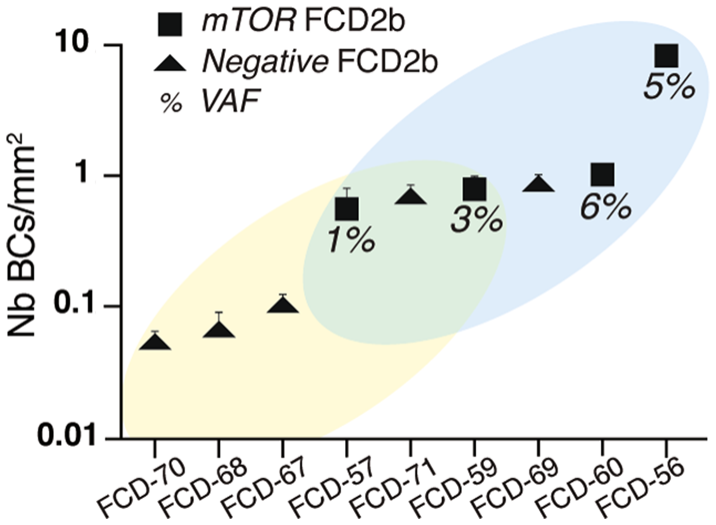
Balloon cells density correlates with genetic findings. Balloon cells density (number of BC/mm^2^) in five panel-negative (yellow region in the plot) and four panel-positive (blue region in the plot) FCD2b cases. The average density calculated on three sections per each patient and the standard error of the mean is reported. The two groups overlap at intermediate density values (green region in the plot)

Regarding mTOR-related FCD2 cases, since somatic variants are mostly present in DNs and BCs, we anticipated that a high density of DNs and BCs present in the sequenced tissue may correlate with an increased likelihood to detect a somatic variant. BCs are reliably recognizable through a standard H&E staining of the frozen brain tissues. To test this hypothesis, we, therefore, counted the density of BCs in five panel-negative and four panel-positive FCD2b cases. We quantified a highly variable density of BCs among FCD2b cases (from 0 to > 400 BCs/section, mean range 0.05 to 8.42 BCs/mm^2^ per patient). Overall, we observed a greater density of BCs in panel-positive compared to panel-negative cases (Fig. 7). However, two panel-negative cases displayed a BCs density above the detection threshold observed (defined by the lowest BCs density compatible with variant detection in our cohort, found in patient FCD-57 with *MTOR*:p.Ser2215Phe at 1% VAF). Besides, in patient FCD-68, we could observe only three BCs in the frozen specimen on average (Fig. 7), while BCs were numerous in the FFPE specimen (Fig. 5b), highlighting possible discrepancy between frozen and FFPE specimen histopathology. Moreover, in patient FCD-21 with FCD2a, who underwent two neurosurgeries, we found an *MTOR* SNV with low VAF (0.59%) and very few DNs in the first surgical specimen, in contrast to a higher VAF (5.7%) and numerous DNs observed from the second surgical specimen, confirming the correlation between the load of pathological cells and the detected VAF.

## Discussion

Pediatric focal epilepsies related to malformations of cortical development (MCDs) are among the most severe epileptic conditions, and surgical resection of the epileptogenic zone is often the only therapeutic option for achieving seizure freedom [15]. In this study, we investigated at the molecular level a large cohort of 80 children with mMCD, FCD, or HME subjected to neurosurgery at a single epilepsy center. We achieved a high molecular diagnostic rate (29% of mMCD/FCD1 cases and 63% of FCD2/HME cases) highlighting the usefulness of clinical genetic testing in these surgical cases.

We provide compelling evidence that mMCD/FCD1 and FCD2/HME are two distinct genetic entities. Brain somatic variants in *SLC35A2* were responsible for 29% of mMCD/FCD1 cases of this cohort. *SLC35A2* encodes a uridine diphosphate–galactose transporter recently involved in a subset of non-lesional intractable focal epilepsies [48] and mild MCDs [45], as well as to X-linked dominant early infantile epileptic encephalopathy-22 (MIM #300896) when the variants are germline [35]. The finding of a non-mTOR-related gene in mMCD/FCD1 is consistent with the associated pathology, characterized by the presence of a focal neuronal migration defect or a focal (and isolated) cortical dyslamination, and the absence of cytomegalic DNs. In contrast, FCD2 and HME cases were exclusively caused by somatic and germline variants in seven mTOR-pathway genes, converging to a common phenotype. Moreover, we show that mTOR hyperactivation occurs similarly in DNs and BCs in both panel-positive and panel-negative FCD2/HME cases, highlighting that FCD2 and HME are all mosaic mTORopathies, even in the absence of a positive genetic diagnosis from a panel of selected genes. Brain mosaicism rates were higher in HME than in smaller FCD2 lesions reflecting the timing of the mutational event and following the same distribution previously reported [10, 29].

Here and in other studies, germline heterozygous loss-of-function variants in repressors of mTOR pathway have been reported in FCD2 (for review [29]). One explanation to sustain how a germline variant affecting a ubiquitous pathway may cause a focal dysplastic lesion is the presence of a somatic second hit, as shown in cancer according to the Knudson’s two-hit model [21] and previously shown in FCD [5, 10, 41]. Here, we demonstrate a somatic second-hit loss-of-heterozygosity (LOH) in an FCD2a patient with a germline *DEPDC5* variant, confirming that biallelic gene inactivation of mTOR repressors underlines the mosaic pathogenesis of FCD. This mosaic LOH mechanism (also reported in TSC patients [30]), has been recently described in one HME patient with a germline *DEPDC5* frameshift deletion [31]. Moreover, this study shows evidence that somatic LOH is mostly restricted to DNs supporting the hypothesis that DNs emerge as a direct consequence of the biallelic loss of *DEPDC5*.

The contribution of DNs and BCs to FCD2-related epileptogenesis has drawn considerable attention and still represents a key question in the field [18]. Evidence from electrophysiological recordings of human FCD2b tissues indicates that BCs are almost silent, while DNs are hyperexcitable [1]. However, their origin remains a mystery. Here, we show that both cell types are the main carriers of the identified variants, indicating that they most likely derive from the same cellular lineage. Overall, these findings led us to postulate that the VAF in bulk tissue is directly correlated with the density of DNs and BCs, with important implications for genetic testing: a low density of these pathological cells can result in a reduced power of variant-detection. Brain somatic mTOR-pathogenic variants were found indistinctly in both FCD2a and FCD2b. The absence of prominent clinical or genetic etiological differences between FCD2a and FCD2b challenge the current classification, so far based only on the presence or absence of BCs, which may not be observed in different brain specimens from the same patient. Whether FCD2a and FCD2b represent truly distinct entities or gradients of the same pathology is indeed a question recently raised by the FCD task force of the ILAE [33].

Here, we performed a systematic investigation of the contribution of somatic and germline variants in a large monocentric cohort of matched fresh frozen and blood samples. We were able to achieve a high genetic diagnostic yield above the one reported in previous studies (between 9 and 41%), elucidating HME cases [10, 19, 27, 31, 32, 45, 48], possibly due to the following reasons. First, we featured a correlation between the density of BCs in the sequenced brain tissue and the variant detection, linking the neuropathological findings to the genetic diagnosis. Second, dedicated sequencing and bioinformatic tools for the detection of low-allele frequency “brain-only” variants were used in this study. Noteworthy, 79% of the reported mutated FCD2 cases have a brain mosaic rate lower than 5%, indicating that the depth of sequencing is critical to achieve high diagnostic yield [29]. Therefore, sequencing at high coverage (at least 1000X to be able to confidently detect VAFs as low as 1%) is crucial to identify somatic variants with low-allele frequencies, and ddPCR can be reliably used to screen for recurrent mutational hotspots and for variant validation. Conventional NGS or Sanger sequencing are unlikely to detect variants occurring at low mosaic levels. Third, the composition of the gene panel is another important parameter: while a small panel comprising only 7 genes of the mTOR pathway could explain 63% of FCD2/HME cases in our cohort, testing *SLC35A2* is recommanded for mMCD/FCD1 cases. This study also confirms the pathogenic role of *RHEB*, a gene recently involved in an HME patient, which should, therefore, be included in MCD/FCD panels [44].

In the mMCD/FCD1 group, however, two-thirds of the cases are panel-negative, with so far only one gene identified, *SLC35A2*, related to glycosylation. A whole exome/genome sequencing approach on matched brain/blood samples is, therefore, necessary to identify novel genes (related or not to glycosylation). These will then be included in dedicated gene panels for the screening of mMCD/FCD1, with an important impact on the development of targeted therapies. Regarding the mTOR-hyperactive FCD2 group, panel-negative cases may be explained by: (1) the lack or insufficient number of variant-carrying pathological cells in the frozen tissue leading to brain mosaicism below the threshold level of detection (2) or by the lack of the causative mTOR-pathway gene in the sequencing panels. Panel-negative FCD2 cases with high densities of DNs or BCs in the tissue available for genetic testing are, therefore, likely to be solved by deep whole exome/genome sequencing approaches from pools of microdissected cells. Moreover, techniques other than capture sequencing (e.g., high-resolution arrays) are necessary to detect small structural variants (such as gene duplications or deletions) or chromosomal rearrangements and intronic variants, that may play a role in the pathogenesis of MCDs. Finally, epigenetic mechanisms such as genomic DNA methylation, that has been recently shown to distinguish FCD subtypes, should also be integrated into the clinicopathologic and molecular assessment of MCDs [22].

Here, we also stressed the absence of correlation between the nature of the mutated gene and the post-surgery outcome, confirming that such conclusions are hampered by multiple factors that are likely to be due to the variability in age at onset, duration of epilepsy, affected cortical area, and by the surgery itself (e.g., hemispherotomy versus focal resection, complete versus incomplete resection). Future larger-scale studies will be needed to improve postsurgical stratifications of patients based on their genetic profile. Yet, routine genetic testing is likely to have future important therapeutic implications in children with intractable epilepsy for potential recruitment to clinical trials. As an example, FCD2 patients for whom surgery and more than two antiepileptic drugs failed to control seizures are enrolled in an ongoing phase-3 clinical trial using the mTOR-inhibitor everolimus (NCT03198949). In *SLC35A2*-related patients, a galactose supplementary diet may improve seizures as previously reported in a case of UDP-galactose transporter deficiency [13]. Recently, groundbreaking results were obtained with the administration of *PIK3CA* inhibitors to patients with CLOVES syndrome, a genetic disorder resulting from somatic gain-of-function variants in *PIK3CA* [47]. HME patients, sometimes associated with CLOVES syndrome, might represent good candidates for PIK3CA inhibitors-based treatment. These novel therapeutic approaches should be considered in patients not eligible for surgery or after an incomplete resection (when seizure freedom is not achieved) or before surgery in patients in which mosaic variants can be detected in peripheral samples (blood, buccal swab or cerebrospinal fluid), as in TSC cases.

## Electronic supplementary material

Below is the link to the electronic supplementary material.

Supplementary file1 (PDF 710 kb)

Supplementary file2 (XLSX 34 kb)
