# Supplementary material for: Dissecting the genetic basis of focal cortical dysplasia: a large cohort study
Source: Acta Neuropathol. 2019 Aug 23;138(6):885–900. doi: 10.1007/s00401-019-02061-5 (PMC6851393; doi:10.1007/s00401-019-02061-5)
Supplement: Supplementary file 1 — Supplementary file1 (PDF 710 kb) [file 401_2019_2061_MOESM1_ESM.pdf]

DISSECTING THE GENETIC BASIS OF FOCAL CORTICAL DYSPLASIA: A LARGE COHORT STUDY

Sara Baldassari PhD[1], Théo Ribierre MSc[1], Elise Marsan PhD[1], Homa Adle-Biassette MD[2,3], Sarah Ferrand-Sorbets MD[4], Christine Bulteau MD PhD[4], Nathalie Dorison MD[4], Martine Fohlen MD[4], Marc Polivka MD[3], Sarah Weckhuysen MD PhD[1,5], Georg Dorfmüller MD[4], Mathilde Chipaux MD PhD[4] and Stéphanie Baulac PhD[1]

1 Sorbonne Université, UPMC Univ Paris 06, UMR S 1127; INSERM, U1127; CNRS, UMR 7225; Institut du Cerveau et de la Moelle épinière (ICM), Hôpital Pitié-Salpêtrière, F-75013, Paris, France.
2 INSERM UMR 1141, Hôpital Robert-Debré, F-75019, Paris, France; Faculté de Médecine Denis Diderot, Université Paris 7, Paris, France.
3 Service d'Anatomie et de Cytologie Pathologiques, Hôpital Lariboisière, APHP, F-75010, Paris, France
4 Department of Pediatric Neurosurgery, Rothschild Foundation Hospital, F-75019, Paris, France.
5 Department of Neurology, University Hospital Antwerp, Belgium.

Corresponding author:
Stéphanie Baulac, PhD
Institut du Cerveau et de la Moelle épinière - ICM
Hôpital Pitié-Salpêtrière - 47, bd de l'hôpital - 75013 Paris
Phone : +33 (0)1 57 27 43 39 / E-mail: stephanie.baulac@upmc.fr

Supplemental Data

Complete list of the genes included in the three versions of the custom panel

Genes reported in blue are common to all versions

Panel version 1 (25 genes): *AKT1* (NM_005163), *AKT2* (NM_001626), *AKT3* (NM_005465), *CNTNAP2* (NM_014141), *DEPDC5* (NM_001242896), *MIOS* (NM_019005), *MTOR* (NM_004958), *NPRL2* (NM_006545), *NPRL3* (NM_001077350), *PIK3CA* (NM_006218), *PIK3CB* (NM_006219), *PIK3CD* (NM_005026), *PIK3R1* (NM_181523), *PIK3R2* (NM_005027), *PIK3R3* (NM_003629), *PTEN* (NM_000314), *RAB3GAP1* (NM_001172435), *SEC13* (NM_183352), *SEH1L* (NM_001013437), *STRADA* (NM_001003787), *TBC1D7* (NM_016495), *TSC1* (NM_000368), *TSC2* (NM_000548), *WDR24* (NM_032259), *WDR59* (NM_030581)

Panel version 2 (30 genes): *AKT3*, *BRAF* (NM_004333.4), *DEPDC5*, *DEPTOR* (NM_022783.3), *FLCN* (NM_144997), *LAMTOR4* (NM_001008395.3), *LARS* (NM_020117.10), *MIOS*, *MLST8* (NM_001199173.1), *MTOR*, *NPRL2*, *NPRL3*, *PIK3CA*, *PIK3R2*, *PTEN*, *RABGAP1L* (NM_014857.4), *RHEB* (NM_005614.3), *RPS6* (NM_001010.2), *RPS6KB1* (NM_003161.3), *RPTOR* (NM_020761.2), *RRAGA* (NM_006570.4), *RRAGAB* (NM_016656.3), *SEC13*, *SEH1L*, *SESN3* (NM_144665.3), *SLC13A5* (NM_177550.3), *TSC1*, *TSC2*, *WDR24*, *WDR59*

Panel version 3 (26 genes): *AKT3*, *BRAF*, *DEPDC5*, *DEPTOR*, *LAMTOR4*, *MIOS*, *MLST8*, *MTOR*, *NPRL2*, *NPRL3*, *PIK3CA*, *PTEN*, *RABGAP1L*, *RHEB*, *RICTOR* (NM_001285439.1), *RPS6*, *RPTOR*, *SEC13*, *SEH1L*, *SLC35A2* (NM_005660.2), *SZT2* (NM_015284.3), *TBC1D7*, *TSC1*, *TSC2*, *WDR24*, *WDR59*

Supplementary Table 2

Droplet digital PCR for detection of VAFs enrichment in DNs and BCs

		MTOR p.Ser2215Phe			MTOR p.Thr1977Lys	PIK3CA p.His1047Arg
	Patient	**FCD-23**	**FCD-24**	**FCD-25**	**FCD-56**	**HME-77**
**VAF (%)**	***Bulk tissue***	4.86 (4.49-5.23)	2.32 (2.03-2.6)	8.73 (8.05-9.41)	3.67 (3.26-4.08)	21.66 (20.92-22.40)
	BCs	N/A	N/A	N/A	45.2 ± 6.7 [15+22]	N/A
	DNs	38.4 ± 2.0 [100+100]	18.6 ± 1.1 [20+20]	37.2 ± 1.1 [80+100]	41.4 ± 20 [25+25]	34.7 ± 6.1 [20+50]
	NNs	7.56 (0.95-14.16) [100]	4.49 (1.15-7.84) [55]	17.98 (10.77-25.19) [100]	1.4 ± 1.9 [50+50]	7.12 (1.46-12.79) [100]
	GCs	8.9 (3.54-14.26) [105]	1.78 (0-5.95) [100]	2.84 (0-6.21) [200]	1.4 ± 2 [50+50]	29.8 (17.8-41.8) [100]

Variant allele frequency (VAF) in the bulk brain DNA and in pools of LCM-isolated cells obtained by ddPCR analysis are reported and the Poisson range (95% confidence interval) is given in parenthesis. When more than one pool was tested per cell type in a patient, the mean VAF ± standard deviation is reported. The number of cells pooled per cell type in each patient is detailed in square brackets (when multiple pools were tested a + is added to separate each pool). BCs: balloon cells, DNs: dysmorphic neurons, NNs: normal appearing neurons, GCs: glial cells.

Supplementary Fig. 1

mTOR activation in FCD2/HME brain specimens

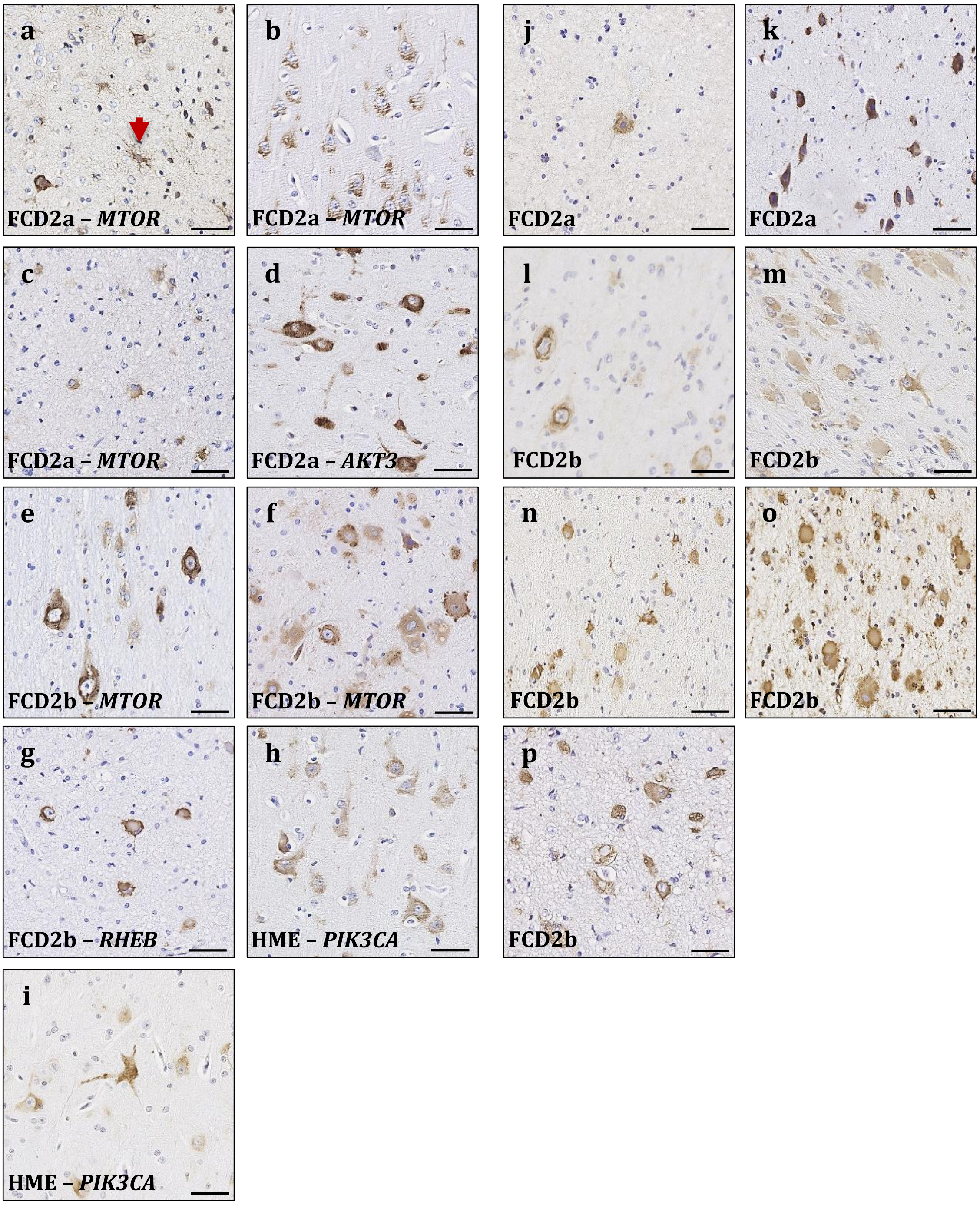

Immunohistochemistry of pS6 on Ser240/244 sites on FFPE 4µm brain sections. pS6 Ser240/244 signal is similar in panel-negative and panel-positive brain samples, suggesting a common pathogenic mechanism (mosaic mTOR hyperactivation). Panel-positive cases: (**a**) FCD-21; (**b**) FCD-26; (**c**) FCD-27; (**d**) FCD-31; (**e**) FCD-57; (**f**) FCD-59; (**g**) FCD-62; (**h**) HME-77; (**i**) HME-78. Panel-negative cases: (**j**) FCD-39; (**k**) FCD-46; (**l**) FCD-67; (**m**) FCD-69; (**n**) FCD-70; (**o**) FCD-71; (**p**) FCD-72. Scale bar: 50µm. In FCD-21 sample, a positive pS6 Ser240/244 signal is detectable in glial cells (red arrow in **a**).
